# Adipose Tissue in Obesity-Related Inflammation and Insulin Resistance: Cells, Cytokines, and Chemokines

**DOI:** 10.1155/2013/139239

**Published:** 2013-12-22

**Authors:** Kassem Makki, Philippe Froguel, Isabelle Wolowczuk

**Affiliations:** ^1^Centre National de la Recherche Scientifique, UMR8199, Lille Pasteur Institute, BP 245, 59019 Lille, France; ^2^University Lille II, 59800 Lille, France

## Abstract

Adipose tissue is a complex organ that comprises a wide range of cell types with diverse energy storage, metabolic regulation, and neuroendocrine and immune functions. Because it contains various immune cells, either adaptive (B and T lymphocytes; such as regulatory T cells) or innate (mostly macrophages and, more recently identified, myeloid-derived suppressor cells), the adipose tissue is now considered as a *bona fide* immune organ, at the cross-road between metabolism and immunity. Adipose tissue disorders, such as those encountered in obesity and lipodystrophy, cause alterations to adipose tissue distribution and function with broad effects on cytokine, chemokine, and hormone expression, on lipid storage, and on the composition of adipose-resident immune cell populations. The resulting changes appear to induce profound consequences for basal systemic inflammation and insulin sensitivity. The purpose of this review is to synthesize the current literature on adipose cell composition remodeling in obesity, which shows how adipose-resident immune cells regulate inflammation and insulin resistance—notably through cytokine and chemokine secretion—and highlights major research questions in the field.

## 1. Adipose Tissue Inflammation Is Crucial in the Development of Obesity-Induced Insulin Resistance

Obesity is a growing epidemic worldwide; its prevalence has been rising tremendously over the last 30 years (WHO, 2013). Excess adiposity is an established risk factor for metabolic diseases including insulin resistance, type 2 diabetes (T2D), hypertension, nonalcoholic fatty liver disease (NAFLD), polycystic ovarian diseases, and several types of cancer [1].

Obesity is a proinflammatory condition in which hypertrophied adipocytes and adipose tissue-resident immune cells (primarily lymphocytes and macrophages) both contribute to increased circulating levels of proinflammatory cytokines. The obesity-associated state of chronic low-grade systemic inflammation, termed “metabolic inflammation,” is considered a focal point in the pathogenesis of insulin resistance and T2D in humans and rodent animal models [2–5]. Although liver and muscle show obesity-induced mild inflammatory responses, white adipose tissue (WAT) is the key site mediating systemic inflammation [6].

### 1.1. Adipose Tissue Promotes an Inflammatory Response in Obesity: Role of TNF-*α*, IL-6, Leptin, Adiponectin, and Resistin in Insulin Resistance

Adipose tissue primary function is to store excess nutrients as triacylglycerols and to release free fatty acids during fasting. A major step forward to the recognition of the major secretory and endocrine role of WAT occurred in the 1990's with the demonstration that adipocytes synthesize and secrete the proinflammatory cytokine tumor necrosis factor alpha (TNF-*α*) [8] and the hormone leptin which regulates appetite and energy balance [9]. Evidence shows that the adipose tissue secretes more than 50 hormones and signaling molecules, collectively called adipokines, which exert their biological roles in an autocrine, paracrine, or systemic manner and influence several physiological processes concerning energy, glucose metabolism, and immunity [10]. More specifically, adipokines can exhibit either proinflammatory or anti-inflammatory properties, thereby contributing to insulin resistance.

Adipose tissue from lean individuals preferentially secretes anti-inflammatory adipokines such as adiponectin, transforming growth factor beta (TGF*β*), interleukin (IL)-10, IL-4, IL-13, IL-1 receptor antagonist (IL-1Ra), and apelin. In contrast, obese adipose tissue mainly releases proinflammatory cytokines among which are TNF-*α*, IL-6, leptin, visfatin, resistin, angiotensin II, and plasminogen activator inhibitor 1 [4]. In lean individuals, anti-inflammatory adipokines mediate physiological functions, whilst in states of metabolic diseases, the proinflammatory adipokines modulate insulin resistance either directly by affecting the insulin signaling pathway or indirectly via stimulation of inflammatory pathways. Indeed, serine phosphorylation of insulin receptor substrate (IRS) by various adipokines directly or via inflammatory pathways including the c-Jun N-terminal kinase (JNK) pathway and I-kappa B kinase *β* (IKK*β*)/NF*κ*B pathway disrupts the insulin signaling pathways, possibly giving rise to insulin resistance [11].

Adipokines enlisted in regulation of insulin resistance are adiponectin, leptin, resistin, visfatin, chemerin, TNF-*α*, IL-1, IL-6, IL-8, IL-10, plasminogen activator inhibitor 1, monocyte chemoattractant protein-1, and retinol binding protein-4 (Tables 1 and 2). Because this topic has been the subject of recent reviews [12, 13] it will not be discussed in detail. We will rather focus on the prototypical adipokines (TNF-*α*, IL-6, leptin, adiponectin, and resistin) highlighting their roles in the development of insulin resistance as well as in immunity and inflammation.

TNF-*α* is a potent proinflammatory cytokine, primarily secreted from myeloid cells via activation of MAPK and NF*κ*B signaling pathways, resulting in the release of other inflammatory cytokines, such as IL-1*β* and IL-6 [14]. It was the first WAT-derived inflammatory cytokine reported to be implicated in the initiation and progression of insulin resistance [8, 15]. Although originally thought to be mainly secreted by adipocytes, it is now admitted that the majority of TNF-*α* is secreted by adipose tissue-resident macrophages [16]. In rodents TNF-*α* is overexpressed in adipose tissue from obese animals, and obese mice lacking either TNF-*α* or its receptor show protection against the development of insulin resistance [17]. In humans TNF-*α* levels are higher in plasma and adipose tissue of obese individuals, and circulating levels reduce with weight loss [18]. TNF-*α* levels were also found to be positively correlated with other markers of insulin resistance [19]; nonetheless, acute treatment with TNF-*α* inhibitor in obese subjects with type 2 diabetes reduced other systemic inflammatory markers without reducing insulin resistance [20], fueling lingering uncertainty about the biological relevance of this pathway in human insulin resistant states. More recently, the long-term assessment of anti-TNF-*α* inhibitor treatment to subjects diagnosed with metabolic syndrome has been shown to improve fasting blood glucose and to increase adiponectin levels, confirming a role for TNF-*α* in obesity-related insulin resistance in humans [21].

A key mechanism by which TNF-*α* induces insulin resistance involved phosphorylation of IRS-1 [22]. Beside its direct negative interference with the insulin signaling pathway, TNF-*α* also indirectly induces insulin resistance by altering adipocyte differentiation and adipocyte lipid metabolism. TNF-*α* is known to promote lipolysis and the secretion of free fatty acids, which contribute to an increase in hepatic glucose production [23]. Moreover, TNF-*α* inhibits the conversion of preadipocytes to mature adipocytes—notably through downregulating adipogenic genes such as peroxisome proliferator-activated receptor gamma (PPAR*γ*) and CCAAT/enhancer binding protein (C/EBP)—allowing further recruitment of uncommitted cells and thus possible expansion of adipose tissue mass [24]. TNF-*α*-activated NF-*κ*B suppressed genes involved in lipid uptake and storage [25] as well as many adipocyte-specific genes. TNF-*α* also downregulates the mRNA levels of adiponectin [26], an adipocyte-derived hormone which contributes to the maintenance of peripheral glucose and lipid homeostasis [27]. Nevertheless, the influence of TNF-*α* on immune response mostly results from its enhancing effect on the production of other cytokines, such as IL-6, rather than from a direct effect.

IL-6 is a multifaceted, pleiotropic cytokine that is a central player in the regulation of inflammation, hematopoiesis, immune responses, and host defense mechanisms [28]. IL-6 is secreted by WAT, skeletal muscle, and liver [16, 29]. Because one-third of circulating IL-6 in healthy individuals is estimated to originate from adipose tissue, IL-6 is considered an adipokine. In WAT, only a fraction of IL-6 is secreted by adipocytes, the other part being produced by other cells, particularly macrophages [16]. Similarly to TNF-*α*, WAT and plasma IL-6 expression correlate with increased body mass, waist circumference, and free fatty acid levels [30], with reduction in circulating IL-6 following weight loss [31]. IL-6 has been implicated as a marker for visceral adiposity because visceral adipose tissue releases more IL-6 than subcutaneous adipose tissue [32]. Nevertheless, data regarding the role of IL-6 in both obesity and insulin resistance are controversial and unresolved. While several studies indicate that increased IL-6 levels correlate with adiposity and fat mass, and not necessarily with insulin action or responsiveness [30, 33], another study has pointed to higher IL-6 levels in patients with obesity-related insulin resistance [34]. It can be inferred that relentless increase in systemic levels of IL-6 may lead to insulin resistance, whereas a transient increase in IL-6 may assist in normal glucose homeostasis. In fact, IL-6 appears to have dual functions depending on the tissue and metabolic state. During exercise, IL-6 increases glucose uptake in the skeletal muscle, leading to muscle hypertrophy and myogenesis and AMPK-mediated fatty acid oxidation, as well as having an anti-inflammatory effect [35]. In adipose tissue and liver, however, IL-6 will exert proinflammatory activities, increasing insulin resistance by upregulating SOCS3 (suppressor of cytokine signaling 3) which, in turn, impairs insulin-induced insulin receptor and IRS1 phosphorylation [36]. IL-6 may promote dysregulation of fatty acid metabolism in WAT as it enhanced mesenchymal stem cell proliferation, maintaining the cells in an undifferentiated state and inhibiting adipogenesis [37]. Additionally, IL-6 was recently shown to stimulate insulin secretion via enhanced GLP-1 (glucagon-like peptide-1) expression in pancreatic cells [38]. Thus, obesity-induced IL-6 secretion may reflect a mechanism to increase insulin production in the obese insulin resistant state. However, while elevated IL-6 secretion from WAT and liver is unfavorable, the opposite is true for skeletal muscle.

On the other hand, a number of *in vitro* and *in vivo* studies demonstrate that IL-6 is capable of inducing insulin resistance. In cultured murine adipocytes, IL-6 production is strongly increased by TNF-*α* and induces insulin resistance by inhibiting glucose uptake and impairing insulin signaling and action [39]. Whether or not IL-6 impairs insulin action in adipose tissue *in vivo* has yet to be clearly determined. Like TNF-*α*, IL-6 can directly affect lipid metabolism and activate pathways to promote increased energy turnover. IL-6 stimulates lipolysis in humans, increases free fatty acid (FFA) concentrations and whole body fat oxidation [40]. Several findings have shown that IL-6 can also affect other adipokines. Notably, IL-6 can decrease the expression and secretion of adiponectin in human adipocytes, as well as other markers of adipocyte differentiation [41]. Overall, IL-6 may play a pivotal role in metabolic diseases, including obesity. Therefore, understanding and clarifying its role in the regulation of metabolism is of utmost importance.

As stated above, leptin was one of the first proteins shown to be secreted from adipose tissue, through the identification and sequencing of the *ob* gene from the *ob*/*ob* mouse [9]. Leptin is primarily secreted by adipocytes proportionally to fat cell mass and is well known for its key contribution to energy metabolism [42]. Leptin exerts its effect on energy balance mainly by acting on the brain, either directly or indirectly by activating specific centers in the hypothalamus to decrease food intake, to increase energy expenditure, to influence glucose and lipid metabolism, or to alter neuroendocrine function. Daily injection of leptin in *ob*/*ob* mice resulted in a rapid reduction in food intake, body mass, and percentage of body fat but maintained lean muscle mass, increased energy expenditure, and restored euglycemia, confirming its important role in energy homeostasis and storage [43]. However, leptin levels are increased in obese subjects, with little or no impact to regulate energy homeostasis, which coined the well-established phrase “leptin resistance” in obesity. Indeed, preclinical and clinical experiments showed that obese rodents and humans displayed leptin resistance that may directly contribute to the reduction of lipid oxidation in insulin-sensitive organs, leading to accumulation of lipids and insulin resistance [44, 45]. Mechanisms leading to leptin resistance are still under investigation. Recently, it has been proposed that SOCS3 could be involved in negative regulation of leptin-induced intracellular signal transduction in the brain [46]. Moreover, neuronal deletion as well as whole-body knock-out of protein tyrosine phosphatase 1B (PTP1B) increased leptin and insulin sensitivity, preventing body weight gain in a diet-induced obesity animal model [47, 48], hence suggesting that, likewise SOCS3, PTP1B also orchestrates leptin resistance control. On the other hand, the role of leptin on insulin resistance is still not fully understood. Leptin is decreased in low insulin states, such as experimentally induced diabetes, and increases after insulin treatment [49]. In humans, insulin resistance is associated with elevated plasma leptin levels independently of body fat mass [50]. However, in patients with lipodystrophy, a condition characterized by almost complete lack of adipose tissue [51], leptin levels are very low and correlate significantly with markers of insulin resistance [52]. Leptin therapy in lipodystrophic patients improves their metabolic state with remarkable improvements in insulin sensitivity, suggesting that leptin acts as a signal that contributes to regulation of total body sensitivity to insulin [53].

Importantly, leptin also plays a key role in controlling immunity and inflammation [54]. Leptin has proinflammatory functions: it stimulates T-cell proliferative responses, polarized naïve CD4^+^ T-cell proliferation towards the Th1 phenotype, promotes a marked increase in Th1-type cytokine production, induces the expression of proinflammatory cytokines by macrophages and monocytes, and acts directly on hepatocytes to promote C-reactive protein expression [55]. The proinflammatory nature of leptin has been noted in several studies, with intravenous injection of endotoxin inducing a sudden rise in leptin levels [56], as well as endotoxin-induced fever and anorexia in rats, again inducing an increase in leptin levels as part of the inflammatory response [57]. The importance of leptin in immunity was confirmed in obese mice with homozygous mutation in *leptin* (*ob*/*ob* mice) or *leptin receptor* (*db*/*db* mice), in which high levels of lymphocyte atrophy and significant reduced thymus cortex were evidenced [58]. Replacement of leptin in the *ob*/*ob* mice or in congenital leptin-deficient children is able to restore normal thymic function, to increase the number of CD4^+^/CD8^+^ T-cells, to promote Th1 differentiation, and to reduce thymic apoptosis. We also reported impaired functionality of T-lymphocytes, dendritic cells, and macrophages in *ob*/*ob* and high-fat (HF) diet-fed mice [59, 60]. More recently, leptin has also been shown to activate human B lymphocytes to secrete TNF-*α*, IL-6, and IL-10 via the JAK2, STAT3, p38MAPK, and ERK signaling pathways [61]. Besides acting on adaptive immunity, leptin also regulates innate immune cells such as polymorphonuclear neutrophils, monocytes, and natural killer (NK) cells [62]. Leptin can induce chemotaxis of neutrophils, is involved in the development and maintenance of a functional NK (natural killer) pool, and induces the production of IL-6 and TNF-*α* from macrophages [55].

Unlike leptin, the circulating levels of adiponectin, a hormone produced predominantly by adipocytes, are decreased in obesity [63]. Adiponectin has important insulin-sensitizing effect: adiponectin-deficient transgenic mouse showed improved insulin sensitivity [64] and association studies have consistently linked plasma adiponectin levels to insulin sensitivity in rodent models and in humans [65]. Among the three major adiponectin isoforms, high-molecular weight (HMW) adiponectin is the most biologically active form and best reflective of the reduction in total adiponectin levels associated with obesity. Indeed, HMW adiponectin levels have been identified as an independent risk factor for insulin resistance [66]. In addition to improving insulin sensitivity, adiponectin exerts anti-inflammatory activity. Adiponectin can suppress the production of TNF-*α* and IFN*γ* (interferon gamma) and is a negative regulator of T cells, notably through its effect on the T-cell presenting function of dendritic cells [67]. Adiponectin maintains a mutual antagonistic action to TNF-*α*: as mentioned above TNF-*α* inhibits the expression of adiponectin [26], and conversely adiponectin suppresses lipopolysaccharide- (LPS-) induced TNF-*α* production [68].

Resistin is another unique adipocyte-derived signaling cysteine-rich molecule that was first identified in obese mice, deriving its name because of its resistance to the action of insulin. In rodents, resistin is secreted primarily from adipose tissue, whereas in humans resistin can be detected in other tissues like placenta, skeletal muscle, small intestine, spleen, stomach, thymus, thyroid gland, and uterus, being predominantly expressed in macrophages [69]. In rodents, initial studies reported increased resistin levels in various models of obesity and insulin resistance [70]. Rajala et al. [71] demonstrated that circulating resistin levels are elevated and positively concordant with rising levels of insulin, glucose, and lipids in *ob*/*ob* mice and that leptin administration improved insulin sensitivity associated with a decrease in *resistin* gene expression. Moreover, transgenic mice overexpressing a dominant negative form of resistin showed increased adiposity, possibly owing to enhanced adipose tissue differentiation and adipocyte hypertrophy [72]. Resistin appears to interfere with normal insulin signaling by decreasing insulin receptor and insulin receptor substrate (IRS1 and 2) protein expression and phosphorylation level in preadipose 3T3-L1 cells [73]. In addition, resistin has been showed to decrease AMPK activation which is known to be implicated as a potential insulin sensitizing molecule [74].

However, the role of resistin in the development of insulin resistance in humans is not as clear as in rodents. Since resistin is preferentially expressed by macrophages in humans, it suggests a proinflammatory role of resistin rather than a role in regulating glucose metabolism. Resistin mRNA expression level is higher in obese subjects, likely resulting from increased infiltration of macrophages in the adipose tissue. Several studies have reported positive correlations between resistin levels and insulin resistance *in vivo* and *in vitro* [70]. Moreover, genetic studies showed that two single nucleotide polymorphisms (SNPs: −537A > C and −420C > G) were associated with increased resistin levels in diabetic patients, but not in control subjects [75]. Recently, associations have been reported between resistin and metabolic syndrome components on one hand and early atherosclerosis in obese children on the other hand [76]. Finally, resistin has been demonstrated to stimulate the secretion of several inflammatory factors (e.g., TNF-*α*, IL-6, IL-8, and MCP-1) known to play a role in the induction of insulin resistance [77]. Therefore, resistin may have an indirect effect on insulin resistance in humans through exacerbating inflammation, which has been shown to disturb insulin sensitivity.

### 1.2. Interleukin-7 Regulates Adipose Tissue Mass and Function

During the past decades, IL-7 has been identified as the major homeostatic cytokine supporting the survival of *αβ* and *γδ* T cells, NKT cells, innate lymphoid cells, and regulatory T cells (Tregs) [78]. IL-7 is predominantly produced by stromal and vascular endothelial cells, with very low levels of *IL7* transcripts detectable in adult animals, consistent with the concept that under basal states there are limited amounts of IL-7 available for lymphocytes *in vivo*. In a homeostatic animal, IL-7 amount is thought to be constant yet stroma-derived IL-7 production can be induced by overt inflammation. IL-7 receptor (IL-7R) is composed of the private IL-7R*α* chain (CD127) combined with the common gamma (*γ*_c_; CD132) chain and is expressed mainly by T lymphocytes but also by NK cells, macrophages, dendritic cells, lymphoid tissue inducer cells, and certain subsets of B cells. One central characteristic of IL-7R expression is its dynamic regulation by cytokines and by the overall metabolic and differentiation state of the cells. For example, TNF-*α* has been reported to upregulate IL-7R*α* expression [79] and IL-6 to be a critical effector of IL-7R signaling [80].

Without IL-7 the lymphoid system cannot be built and maintained. Interestingly, the role of IL-7 on lymphocyte homeostasis was shown to partly rely on its control of basal lymphocyte glucose metabolism through the expression of the glucose transporter GLUT-1, which promotes glucose uptake and increases metabolic activity as well as cell size [81].

Recently, we and others identified IL-7 as a new secretory product of the adipose tissue, mostly produced by cells of the stromal vascular fraction [82, 83]. Furthermore, we reported that IL-7 also contributes to body weight regulation *via *both hypothalamic [84] and adipose tissue [82] control. Regarding the latter, we showed that IL-7 modulates the adipose tissue through acting on its mass and function. In fact, a single administration of IL-7 was sufficient to decrease adipose tissue inflammation and to protect mice from obesity in three different models of experimentally induced obesity (i.e., monosodium glutamate-induced hypothalamic obesity [84], gold thioglucose-induced hypothalamic obesity (Wolowczuk I, unpublished data), and HF diet- (HFD-) induced obesity [82]).

Strikingly, we showed that IL-7 overexpressing mice presented a lipodystrophy-like phenotype: reduced WAT mass is associated with impaired adipocyte differentiation and intolerance to glucose and insulin resistance, these traits being commonly associated with lipodystrophy in both animals and humans [51].

## 2. Adipose Tissue Cellular Remodeling in Obesity

The first part of our review showed that the apparent metabolic simplicity of the adipose tissue is illusory; this is also true regarding its cellular composition. Besides lipid-filled mature adipocytes, the tissue is also composed of various stromal cells, including preadipocytes, endothelial cells, fibroblasts, and immune cells [13]. During the progression of obesity, both the adipocyte and the stroma vascular fractions are changed: adipocytes grow larger, secrete predominantly proinflammatory cytokines, and are insulin resistant; coincidently, the nature of WAT immune cells is also modified.

### 2.1. Changes in Immune Cell Composition in the Obese Adipose Tissue: A Focus on MCP-1 and CCR5 Chemokines

Obesity is characterized by the accumulation of diverse immune cells in the adipose tissue. Notably, proinflammatory macrophage infiltration and inflammation-related gene expression precede the development of insulin resistance and appear to be a cardinal feature of obesity in rodents and humans [16].

Adipose tissue macrophages (ATMs) accumulate in both the subcutaneous and visceral expanding fat depots, even though macrophage infiltration appears to be more prominent in the latter [86]. Apart from increasing in numbers, adipose tissue macrophages are also phenotypically changed during obesity: while anti-inflammatory M2 macrophages reside in WAT of lean mice, obese WAT predominantly contains proinflammatory M1 macrophages [87]. Activated M1 ATMs are a prominent source of proinflammatory cytokines such as TNF-*α* and IL-6, which can block insulin action in adipocytes via autocrine/paracrine signaling causing systemic insulin resistance via endocrine signaling (cf.  Section 1). Thus, both recruitment and proinflammatory polarization of ATMs are required for the development of insulin resistance. In both humans and rodents, ATMs content positively correlates with inflammation and insulin resistance [16]. Despite its importance in adipose tissue inflammatory responses and systemic insulin sensitivity, the mechanisms underlying M1 versus M2 macrophage polarization still remain poorly understood. The recent discovery of microRNAs (miRNAs) provides a new opportunity to understand this complicated but crucial network for macrophage activation and adipose tissue function. miRNAs, which correspond to a group of highly conserved, small (i.e., approximately 22 nucleotides in length) noncoding RNAs, can trigger either a block in translation and/or mRNA degradation [88, 89]. Numerous studies have provided compelling evidence that miRNAs are key regulators of cell fate determination and significantly contribute to the pathogenesis of complex diseases, including obesity-associated metabolic diseases [90–92]. Zhuang et al. [93] recently identified miRNA-223 (miR-223) as a potent regulator of macrophage polarization and provided strong evidence supporting the functional significance of this new pathway in metabolic homeostasis. The authors showed a suppressive effect of miR-223 on macrophage proinflammatory activation (M1) and a stimulatory effect on anti-inflammatory activation (M2): high-fat diet-fed miR-223-deficient mice displayed increased adipose tissue inflammation and were more insulin resistant. At the molecular level, a major target of miR-223 in macrophages is Pknox1, which itself favors the proinflammatory activation pathway. However, a key question still unanswered by now is how the miR-223/Pknox1 pathway interacts with known regulatory pathways that control macrophage activation. The identification of mechanisms underlying functional polarization of macrophages into M1 or M2 might provide new insights into a basis for macrophage-centered therapeutic strategies for metabolic diseases.

Similarly to any immune and inflammatory response, macrophage infiltration in the obese adipose tissue results from blood monocyte influx, mainly attracted by the chemokine monocyte chemoattractant protein-1 (MCP-1) which is secreted by hypertrophic adipocytes. It has been reported that MCP-1 secretion is markedly enhanced locally and in plasma of obese rodents and humans [94]. Overexpression, deficiency, or mutation-induced dysfunction of MCP-1 in different mouse models were shown to interfere with ATMs accumulation, along with insulin-resistance development [95, 96]. However, the role of MCP-1 in promoting ATM recruitment and insulin resistance has recently been challenged by the absence of noticeable impact on macrophage accumulation and glucose intolerance resulting from MCP-1 genetic disruption [97]. Furthermore, HFD-fed MCP-1 receptor i.e., CCR2-deficient mice (namely, *ccr2* ^−/−^ mice) do not normalize ATM content and insulin resistance to the levels of lean animals [96], suggesting that ATM recruitment and insulin resistance are also regulated by MCP-1/CCR2 independent signaling pathways.

Kitade et al. recently identified and characterized a critical role for CCR5, another C-C motif chemokine receptor, in the regulation of obesity-induced WAT inflammatory response and insulin resistance [98]. These authors reported accumulation of CCR5 expressing ATMs in HFD-fed mice. Importantly, *Ccr5* ^−/−^ mice were protected from insulin resistance induced by HF feeding through both reduction in ATM accumulation and induction of anti-inflammatory M2 shift in those cells [98]. Additionally, a bone marrow transplantation study revealed that lack of CCR5 expression in macrophages alone could protect mice from the HFD-induced insulin resistance, this being associated with a significant reduction in ATM infiltration. In humans, recent studies have also shown upregulation of CCR5 in the visceral fat of morbidly obese individuals in whom macrophage infiltration has been confirmed [99]. However, further studies are needed to evaluate whether CCR5 inhibitor treatment (e.g., maraviroc) affects macrophage activation and other aspects of adipose tissue biology in obese patients. Also, it remains to be established whether the two C-C chemokine receptors, CCR2 and CCR5, play common or unique roles in obesity-induced adipose tissue inflammation and insulin resistance.

Alterations in ATM content and polarization state occur fairly late in the progression of obesity and probably are not initiating events of inflammation and development of sustained insulin resistance. Evidence has accumulated showing that other changes in adipose-resident immune cells may precede these events. Under this scenario, ATMs will be effectors of a coordinated inflammatory response that includes the accumulation of proinflammatory T cells (CD8^+^ and Th1 CD4^+^ T cells) and the loss of anti-inflammatory regulatory T cells (Tregs), as well as the appearance of B cells, NK cells, NKT cells, eosinophils, neutrophils, and mast cells [100].

Adipocytes in lean adipose tissue produce factors such as IL-4 and IL-13 that induce M2 activation of macrophages and Th2 activation of CD4^+^ T cells and maintain Treg cell and eosinophil numbers. In obesity, the progressive accumulation of adipose tissue is accompanied by early increased infiltration of proinflammatory CD8^+^ T cells and a shift towards a higher CD8^+^/CD4^+^ ratio. CD8^+^ infiltration appears to be a key event preceding the depletion of adipose Tregs and the increased CD4^+^ Th1 cell activation observed in murine models of diet-induced obesity [101, 102]. Increased adipose-resident CD8^+^ T-cell activation also potentiates adipocyte expression of IL-6 and TNF-*α* in mice, while CD8^+^ T-cell depletion reverses this effect.

Neutrophils are known to play a role in the early stages of inflammatory responses, and it has been recently reported a sustained increased in adipose tissue neutrophil content in HFD-induced obesity with neutrophil secreted elastase being a key effector in this process [103]. The enhanced release of IL-8, a factor involved in neutrophil chemotaxis, by hypertrophic adipocytes, may partly explain neutrophil recruitment. Adipose tissue neutrophils produce chemokines and cytokines, facilitating macrophage infiltration, which could contribute to development of insulin resistance.

### 2.2. Myeloid-Derived Suppressor Cells: A Novel Actor in the Control of Insulin Sensitivity

In the 1980s, a new cell population known as “natural suppressor cells,” distinct from T and NK cells, was described in the bone marrow and spleen of tumor-bearing mice [104, 105]. Later on, these cells were defined as “myeloid-derived suppressor cells” (MDSC) because of their myeloid origin and their ability to suppress immune responses [106]. In fact, MDSCs represent a heterogeneous and metabolically plastic population of immature myeloid cells in different stages of differentiation, having in common the capacity to inhibit effector immune responses and to accumulate under conditions of inflammation [107]. The term plasticity here refers to the ability of MDSCs to change both their expression of various mediators of suppression (e.g., iNOS, arginase I) in response to environmental influences (e.g., local IL-4/13 or IFN*γ* concentration) and also their differentiation state (e.g., becoming more/less neutrophil or myeloid cells). These cells are also defined by their immature state of macrocytic/monocytic, granulocytic/neutrophilic, and dendritic cell precursors and are characterized by the increased production of extracellular degradative enzymes, cytokines, and reactive oxygen and nitrogen species [108].

In mice, MDSC are commonly identified as coexpressing the cell surface markers CD11b and Gr-1. Since there are several subpopulations within Gr-1^+^CD11b^+^ cells, several groups further subcategorized MDSC into “monocytic” MDSC (CD11b^+^Ly6G^−^Ly6C^high^) and “granulocytic/neutrophil-like” MDSC (CD11b^+^Ly6G^+^Ly6C^low^), based on the expression of Ly6C and Gr-1/Ly6G [109]. There is no human marker equivalent to mouse Gr-1, human MDSC being typically defined as CD11b^+^CD33^+^CD34^+^CD14^−^HLA-DR^−^ cells [110]. In addition to heterogeneity, discrepancies exist in cell surface expression of certain activation/maturation markers, such as MHC II and costimulatory molecules, and of lineage markers (e.g., F4/80) between MDSC. This heterogeneity supports the notion that MDSC include multiple subpopulations of myeloid-derived cells that are at various stages of maturity.

In the steady state, MDSCs are predominantly present in the bone marrow and participate in the normal process of myelopoiesis. However, under various pathological inflammatory conditions such as cancer, infection, sepsis, graft-versus-host disease, and bone marrow transplantation, a variety of cytokines and soluble factors released induce rapid expansion of MDSC that will accumulate in peripheral lymphoid organs and blood, as well as in tumors, where they have been described to block CD4^+^ and CD8^+^ T-cell responses thus favoring cancer development [111]. In cancer, one key factor controlling MDSC expansion and tumor progression is PPAR*γ* [112]. Vascular endothelial growth factor (VEGF), macrophage colony-stimulating factor (M-CSF), or IL-6 is also required for MDSC expansion. It has been suggested that MDSCs contribute to tumor progression by both facilitating neoangiogenesis and metastasis [113] and by inhibiting antitumor responses [111]. In addition to their recognized role in tumor tolerance, MDSCs may also be involved in the induction and maintenance of transplant tolerance [114].

Recently, an exciting observation has been described by Xia et al., showing that MDSCs and M2 macrophage induction may be a physiological response to promotion of insulin sensitivity [115]. These authors showed that obese *ob*/*ob* mice, as well as wild-type mice fed on high-fat diet, have marked accumulation of anti-inflammatory MDSCs and M2 macrophages in adipose tissue. Furthermore, the increase in MDSCs and M2 macrophage number was associated with higher response to insulin. Adoptive transfer of MDSCs (e.g., Gr-1^+^ cells) into high-fat diet-fed mice improved the response of the recipient mice to insulin while, in contrast, MDSCs depletion (after treatment with anti-Gr-1 antibody) increased their susceptibility to obesity and further worsened their resistance to insulin. Yin et al. have also described the ability of MDSCs to delay onset of type 1 diabetes and insulin resistance [116], through inducing expansion of antigen-specific Tregs and suppressing T-cell proliferation. The mechanisms by which obesity-associated chronic inflammation induces expansion/accumulation of MDSCs are arguably stepwise. However, the initial proinflammatory state created in early obesity may induce the accumulation of MDSC in an attempt to curtail overt inflammation, as described in other well-described models of inflammation [107], and may improve insulin sensitivity. In addition, we recently showed that the mechanistic target of rapamycin (mTOR) signaling pathway might be involved in the expansion of MDSCs (Makki, MS submitted), as well as in myelopoiesis [117]. Although mechanisms by which MDSCs enhance insulin sensitivity are unknown, it has been proposed that upregulation of insulin growth factor-1 (IGF-1) in the setting of insulin resistance may lead to the accumulation of MDSCs or M2 macrophages [118]. Suggestions have been made that not only does insulin resistance induce physiological response for MDSC and M2 macrophage expansion, but insulin may also modulate direct gene transcriptional control of these cells. Therefore, pharmacological enhancement of insulin sensitivity in obese individuals may preemptively hinder the development of MDSCs.

## 3. Concluding Remarks

**Figure 1:**
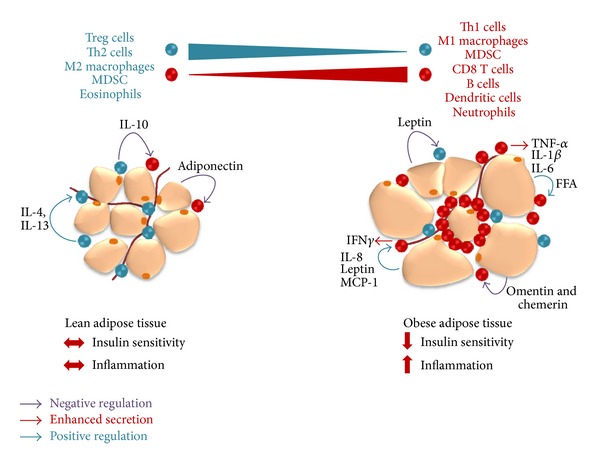
Adipose tissue-resident cells, cytokines, and hormones: role in insulin sensitivity (adapted and updated from [7]).

The complex alterations in adipose tissue secretion of cytokines, adipokines, and chemokines and immune cell composition observed in adipose tissue-related pathologies such as obesity (Figure 1) have been, and still are, an active research area. As the proportion of overweight and obese (even among the youngest) continues to rise worldwide, understanding the role of adipose tissue in the pathogenesis of obesity and its metabolic and immune-based complications will be critical to optimize long-term health outcomes. As summarized in the present review, there might be a potential therapeutic value of targeting certain immune resident cells (such as M2, Tregs, or MDSC) and/or certain cytokines, adipokines, or chemokines (such as MCP-1/CCR2 or CCR5) to improve insulin resistance and restrain organ damage in type 2 diabetic obese patients by limiting the proinflammatory milieu.

## Figures and Tables

**Table 1:** Adipokines increased in obesity and/or diabetes (adapted and updated from [85]).

Adipokine	Distribution	Function	Increased in obesity
Leptin	Secreted predominantly by WAT, to a lesser degree, in hypothalamus, gastric epithelium, placenta, and gonads	Regulates energy intake, expenditure and feeding behavior. Also regulates storage of fat and insulin signaling	Increased in mouse models of obesity. Increased in human obesity and correlated with BMI and decreased with weight loss

Resistin	In rodents, secreted by adipocytes. In humans, secreted predominantly by circulating macrophages and monocytes, to a lesser degree, by WAT	Implicated in glucose metabolism, in the regulation of neoglucogenesis and insulin resistance in rodents. More proinflammatory role in humans	Increased circulating concentrations in mouse models of obesity. Increased in human obesity and correlated with insulin resistance in diabetic patients

TNF-*α*	Expressed by macrophages and adipocytes (visceral WAT > subcutaneous WAT)	Affects insulin and glucose metabolism. Provokes insulin resistance and stimulates lipolysis	Increased in mouse models of obesity. Increased in human obesity and correlated with BMI

IL-6	One-third of total circulating levels are expressed predominantly by adipocytes. Also expressed in macrophages, skeletal muscle, endothelial cells, and fibroblasts	Controversial role in the development of insulin resistance. Affects glucose metabolism	Increased circulating levels in human obese subjects and correlated with adiposity and reduced with weight loss. Increased in plasma of T2D patients

IL-7	Secreted by stromal and vascular endothelial cells	Homeostatic immune cytokine. Also regulates body weight, adipose tissue mass and function, and insulin signaling	Increased in morbidly obese subjects

IL-8	Secreted by adipocytes (visceral WAT > subcutaneous WAT) and macrophages	Neutrophil chemotaxis	Increased in obese subjects and related to fat mass and TNF-*α* levels

IL-1	Secreted mainly by adipocytes and macrophages	Role in macrophages chemotaxis and thermogenesis	Increased in obese mice. Increased in human obesity and predictive of T2D

RBP4	Secreted by adipocytes, macrophages, and hepatocytes	Affects insulin sensitivity, hepatic glucose output, and muscle insulin signaling	Increased circulating levels in obese subjects and correlated with BMI and insulin resistance

MCP-1	Secreted by adipose tissue	Affects insulin sensitivity and increases macrophage recruitment in adipose tissue and inflammation	Increased in mouse models of obesity. Increased in T2D subjects

PAI-1	Expressed by WAT	Potent inhibitor of fibrinolytic pathway	Increased in human obesity and T2D subjects

CXCL5	Secreted by macrophages within the stromal vascular fraction	Interferes with insulin signaling in muscle	Circulating levels are higher in obese insulin-resistant individuals than in obese insulin-sensitive and decreased after a 4-week period on low-calorie diet

Visfatin	Expressed in liver, muscle, WAT, bone marrow, and lymphocytes	Role in insulin sensitivity, insulin secretion and inflammatory properties	Increased in obesity and correlates with visceral adiposity in humans

Chemerin	In rodents and humans, expressed in placenta and WAT	Regulates adipocyte development and metabolic function	Increased circulating levels in obese and T2D patients and correlated with body fat, glucose, and lipid metabolism

Vaspin	Secreted by WAT, hypothalamus, pancreatic islets, and skin	Improves insulin sensitivity	Increased in obesity and T2D patients

**Table 2:** Adipokines decreased in obesity and/or diabetes (adapted and updated from [85]).

Adipokine	Distribution	Function	Decreased in obesity
Adiponectin	Only secreted by adipose tissue. Lower production in men	Insulin sensitizing effect. Improves insulin resistance and glucose metabolism	Decreased in mouse models of obesity. Decreased in human obesity and correlated negatively with BMI. Increased after weight loss

IL-10	Secreted by monocytes, macrophages, dendritic cells, and B and T cells	Improves insulin sensitivity and glucose transport	Attenuated in T2D patients and increased with weight loss

Omentin	Expressed in heart, lungs, ovary, and placenta and predominantly produced by WAT	Improve glucose uptake in human adipocytes and has an anti-inflammatory effect	Decreased circulating levels in obese subjects. In impaired glucose tolerant (IGT) and subjects with T2D, circulating levels are lower those when compared with matched controls
